# Panspecies Small-Molecule Disruptors of Heterochromatin-Mediated Transcriptional Gene Silencing

**DOI:** 10.1128/MCB.01102-14

**Published:** 2015-01-23

**Authors:** Emilie Castonguay, Sharon A. White, Alexander Kagansky, Daniel J. St-Cyr, Araceli G. Castillo, Christiane Brugger, Rachel White, Carolina Bonilla, Michaela Spitzer, William C. Earnshaw, Thomas Schalch, Karl Ekwall, Mike Tyers, Robin C. Allshire

**Affiliations:** a Wellcome Trust Centre for Cell Biology, Institute of Cell Biology, School of Biological Sciences, The University of Edinburgh, Edinburgh, United Kingdom; b MRC Human Genetics Unit, Institute of Genetics and Molecular Medicine, The University of Edinburgh, Edinburgh, United Kingdom; c Institute for Research in Immunology and Cancer, Department of Medicine, Université de Montréal, Montréal, Québec, Canada; d Instituto de Hortofruticultura Subtropical y Mediterránea La Mayora, Universidad de Málaga, Consejo Superior de Investigaciones Científicas (IHSM-UMA-CSIC), Area de Genética, Campus de Teatinos, Málaga, Spain; e Department of Molecular Biology, Science III, Institute of Genetics and Genomics of Geneva (iGE3), University of Geneva, Geneva, Switzerland; f Department of Biosciences and Nutrition, Karolinska Institutet, Novum, Huddinge, Sweden

## Abstract

Heterochromatin underpins gene repression, genome integrity, and chromosome segregation. In the fission yeast Schizosaccharomyces pombe, conserved protein complexes effect heterochromatin formation via RNA interference-mediated recruitment of a histone H3 lysine 9 methyltransferase to cognate chromatin regions. To identify small molecules that inhibit heterochromatin formation, we performed an *in vivo* screen for loss of silencing of a dominant selectable *kanMX* reporter gene embedded within fission yeast centromeric heterochromatin. Two structurally unrelated compounds, HMS-I1 and HMS-I2, alleviated *kanMX* silencing and decreased repressive H3K9 methylation levels at the transgene. The decrease in methylation caused by HMS-I1 and HMS-I2 was observed at all loci regulated by histone methylation, including centromeric repeats, telomeric regions, and the mating-type locus, consistent with inhibition of the histone deacetylases (HDACs) Clr3 and/or Sir2. Chemical-genetic epistasis and expression profiles revealed that both compounds affect the activity of the Clr3-containing Snf2/HDAC repressor complex (SHREC). *In vitro* HDAC assays revealed that HMS-I1 and HMS-I2 inhibit Clr3 HDAC activity. HMS-I1 also alleviated transgene reporter silencing by heterochromatin in Arabidopsis and a mouse cell line, suggesting a conserved mechanism of action. HMS-I1 and HMS-I2 bear no resemblance to known inhibitors of chromatin-based activities and thus represent novel chemical probes for heterochromatin formation and function.

## INTRODUCTION

Specialized chromatin domains termed heterochromatin are important for mediating dosage compensation, monoallelic imprinting, and cell lineage-specific gene expression. Large heterochromatin domains are associated with arrays of repetitive elements found at centromeres in many eukaryotes (1). Such heterochromatic regions in most genomes tend to be devoid of genes, and the transcription of genes placed within heterochromatin is inhibited because the resident repetitive elements attract chromatin-modifying activities that repress transcription (2, 3). Transcriptionally repressive modifications such as H3K9 methylation (H3K9me) are prevalent in heterochromatic regions, whereas activating modifications, such as histone acetylation, are scarce (4, 5). H3K9 methylation allows the binding of specific chromodomain proteins, including HP1 (heterochromatin protein 1), which recruit a variety of key chromatin-modifying activities (6–8). Heterochromatin formation on repetitive elements renders these regions transcriptionally inert and promotes genome stability through the regulation of recombination, DNA repair, and chromosome segregation (3). In fungi, plants, and animals, the integrity of heterochromatin can be monitored by the use of transcriptionally silent reporter genes placed within or close to centromeric repeats or elsewhere (9–11).

In the fission yeast Schizosaccharomyces pombe, heterochromatin is found at centromeres, telomeres, and the silent mating-type locus. Many of the key components involved in forming heterochromatin are conserved in multicellular eukaryotes (12). Clr4 methyltransferase is the fission yeast counterpart of metazoan Suv39; the enzyme methylates histone H3 specifically on lysine 9 (H3K9me) (4, 13). H3K9 methylation creates a binding site for the chromodomain proteins Swi6 and Chp2, which are the orthologs of metazoan HP1 (6, 7, 14). RNA interference (RNAi) utilizes the conserved core activities of Dicer (Dcr1), Argonaute (Ago1), and RNA-dependent RNA polymerase (Rdp1) to direct Clr4 methyltransferase to specific chromatin regions in fission yeast (12). The binding of Swi6 and Chp2 to H3K9me results in the recruitment of the Snf2/histone deacetylase (HDAC) repressor complex (SHREC) to complete heterochromatin formation and transcriptional silencing (15, 16). SHREC consists of the HDAC Clr3, the SNF2-related chromatin remodeling factor Mit1, and two nonenzymatic components, Clr1 and Clr2 (15). The HDACs Clr6 and Sir2 also facilitate removal of histone acetylation to maintain heterochromatin integrity (17–19). SHREC resembles the vertebrate Mi-2/NuRD silencing complex, which contains HDAC1/2 and SNF2 ATPase Mi-2, among other proteins (20), although it remains unclear whether RNAi directly modulates chromatin modification in mammals (1). Related protein complexes are known to couple RNAi to DNA methylation, H3K9 methylation, and gene silencing in plants (21, 22). In Arabidopsis, RNAi also contributes to heterochromatin integrity by directing *de novo* DNA methylation to homologous sequences (23, 24), where it recruits Suv39 methyltransferase related proteins (25).

RNAi and heterochromatin components are not essential for viability of fission yeast. This has facilitated mechanistic dissection of the process initially through genetic screens and subsequently via mass spectrometric analysis of purified protein complexes (10, 15, 26–29). Deletion of individual RNAi or heterochromatin components disrupts silencing of reporter genes inserted within heterochromatin (10, 15, 28, 30). Small-molecule inhibitors provide an alternative means for probing biological pathways. In contrast to mutations, inhibitor effects are usually reversible and thereby enable precise determination of functional dependencies in complex pathways (31–33). For example, *in vivo* screens based on telomere position effect in budding yeast have previously allowed the identification of sirtinol and splitomicin, which inhibit Sir2 (34, 35). Fission yeast is amenable to high throughput cell-based screens (36–38) and the integrity of its heterochromatin and associated gene silencing have been shown to be sensitive to the HDAC inhibitor trichostatin A (TSA) (39, 40). Unbiased small-molecule screens may thus identify novel compounds that inhibit the function of components of the RNAi-directed chromatin modification system in fission yeast, such as Dicer, Argonaute, Clr4 H3 lysine 9 methyltransferase and the various HDACs. Because small molecules identified from yeast screens may also inhibit conserved orthologs (41–44), inhibitors of fission yeast heterochromatin integrity may yield insights into related processes in higher eukaryotes, including humans. Small-molecule inhibitors of heterochromatin may be of therapeutic value in cancer and other diseases caused by aberrant gene regulation. For example, the HDAC inhibitors vorinostat and romidepsin, as well as the histone lysine methyltransferase inhibitor chaetocin, have antitumorigenic activity (45, 46).

We report here a cell-based screen for small-molecule inhibitors of fission yeast heterochromatin. Two novel compounds, called HMS-I1 and HMS-I2, were identified that disrupt heterochromatin integrity at the level of the SHREC complex. HMS-I1 also disrupts transgene silencing in the plant Arabidopsis thaliana and in mammalian cells. Both compounds appear to exert their effect on heterochromatin integrity through inhibition of class II HDACs. This screen in fission yeast has thus identified novel small molecules that interfere with heterochromatin integrity across the fungal, plant, and animal kingdoms.

## MATERIALS AND METHODS

### Fission yeast growth and chemical screens.

Haploid Schizosaccharomyces pombe cells were grown in YES (yeast extract with supplements) medium at 32°C and assessed in log phase for all experiments. Cells and compounds were dispensed in 96-well microplates using a Biomek FX liquid handling robot (Beckman Coulter) and plates were read (optical density at 595 nm [OD_595_]) every 15 min for 48 to 72 h at 32°C with continuous shaking in a Sunrise plate reader (Tecan). Growth curves generated for each compound were analyzed using in-house R scripts and the grofit R package to extract parameters for doubling time, lag time and saturation time. Table 1 contains a list of the strains used in the present study.

**TABLE 1:** Fission yeast strains used in this study

Strain	Genotype
1056	*mat1*::*ura4 ade6-210 leu1-32 ura4^−^*
5962	*h^−^ clr3*::*kanMX ade6-210 ura4-D18 leu1-32*
8878	*h^−^ sir2*::*NatMX ade6-210 leu1-32 arg3-D4 his3-D ura4-D18*
9299	*h90 mat3*::*ura4^+^ ura4-DS/E dcr1D*::*NAT*
9351	*h^+^ sir2*::*NatMX his3D arg3-D4 leu1-32 ade6-210 ura4-D18*
17425	*h^+^ dcr1*::*KanMX otr1R*(SphI):*ade6^+^ ade6-210 leu1-32 ura4-D18*
17668	*h^−^ dcr1*::*KanMX sir2*::*NatMX ade6-210 leu1-32 ura4-D18*
17860	*h^90^ dcr1*::*KanMX sir2*::*NatMX ade6-210 leu1-32 ura4-D18 his3-D1*
18618	*h? clr3*::*KanMX sir2*::*NatMX ade6-704–HygMX6 leu1-32 ura4-D18*
19650	*Mat1-MsmtO leu1-32 ade6-210 his2 ura4-DS/E clr3-3×FLAG-KanMX otr1R*(SphI)::*ura4^+^*
20079	*h^+^ ade6-210 leu1-32 ura4-D18 imr1R* (*dg-glu*) NcoI::*kanMX oriII*
20080	*h^−^* (XbaI-SpeI)*clr4*::*LEU2 ade6-210 leu1-32 ura4-D18 imr1R* (*dg-glu*) NcoI::*kanMX oriII*
20303	*h^+^ clr2*::*his7^+^ ade6-216 ura4-D18 his7-366 leu1-32*
20304	*h^+^ clr2*::*kanMX ade6-210/216 ura4-D18 leu1-32*
20305	*h^+^ mit1*::*kanMX ade6-210 ura4-D18 leu1-32*
20306	*h^−^ mit1*::*kanMX ade6-210 ura4-DS/E leu1-32*
SPT429	*Mat1-MsmtO leu-132 ade6-210 his2 ura4-DS/E mit1-13*×*myc-KanMX otr1R*(SphI)::*ura4^+^*

### Characterization of chemical compounds.

^1^H and ^13^C NMR, recorded at 400 and 100 MHz, respectively, were performed on a Varian 400-MR spectrometer. Assignments of ^1^H and ^13^C NMR signals were carried out by correlation spectroscopy (COSY) and Heteronuclear Single Quantum Coherence (HSQC) experiments. Mass spectral (MS) and high-resolution mass spectrometry (HRMS) data were recorded on Agilent 6120 Quadrupole LC/MS and Agilent LC/MSD TOF (model 61969A) systems, respectively.

The data obtained for compound 2-(2,3-dihydrobenzo[*b*][1,4]dioxin-6-yl)-6-methylimidazo[1,2-*a*]pyridine (HMS-I1) were as follows: (i) ^1^H NMR (400 MHz, CDCl_3_): δ (ppm) 7.87 (s, 1H, MeCCHN), 7.66 (s, 1H, NCHC), 7.50 (d, *J* = 9.00 Hz, 1H, N_2_CCH), 7.41 to 7.47 (m, 2H, *o*-CHCC), 6.99 (dd, *J* = 1.17, 9.39 Hz, 1H, MeCCHC), 6.92 (d, *J* = 7.83 Hz, 1H, *p*-OCCH), 4.30 (s, 4H, OCH_2_), 2.31 (s, 3H, CH_3_); (ii) ^13^C NMR (100 MHz, CDCl_3_): δ (ppm) 145.4, 144.8, 143.9, 143.6, 127.8, 127.7, 123.4, 122.0, 119.4, 117.6, 116.8, 115.0, 107.3, 64.6, 64.5, 18.2; (iii) ^13^C NMR (100 MHz, CDCl_3_, CH_(1/3 or 2)_ from HSQC): δ (ppm) 127.7 (MeCCHC), 123.4 (MeCCHN), 119.4 (*p*-OCCCH), 117.7 (*p*-OCCH), 116.8 (N_2_CCH), 115.1 (*m*-OCCH), 107.3 (NCHC), 64.5 (OCH_2_), 18.2 (CH_3_); (iv) HRMS (ESI-TOF) *m*/*z*: [M + H]^+^ calculated for C_16_H_15_N_2_O_2_ 267.1128; found 267.1137.

The data obtained for compound *N*-(benzo[*b*]thiophen-2-yl)-4-(2-chloro-6-fluorobenzyl)piperazine-1-carboxamide (HMS-I2) were as follows: (i) ^1^H NMR (400 MHz, CDCl_3_): δ (ppm) 7.72 (d, *J* = 7.92 Hz, 1H), 7.55 (d, *J* = 7.63 Hz, 1H), 7.13 to 7.32 (m, 4H), 6.96 to 7.06 (m, 2H, ArCH, NH), 6.73 (s, 1H), 3.76 (d, *J* = 2.35 Hz, 2H, CH_2_Ar), 3.45 to 3.59 (m, 4H, CH_2_NCO), 2.59 to 2.66 (m, 4H, CH_2_NCC); (ii) ^13^C NMR (100 MHz, CDCl_3_, CH_(1/3 or 2)_ from HSQC): δ (ppm) 129.4 (CH), 125.6 (CH), 124.3 (CH), 122.4 (CH), 121.8 (CH), 121.5 (CH), 114.0 (CH), 105.2 (CH), 52.3 (CH_2_Ar), 52.2_8_ (CH_2_NCC), 44.2 (CH_2_NCO); (iii) LC-MS (APCI) *m*/*z*: [M+H]^+^ calculated for C_20_H_20_ClFN_3_OS 404.1; found 404.1.

### ChIP.

Chromatin immunoprecipitation (ChIP) analysis was performed as previously described (47). Briefly, cells were grown at 32°C to log phase and fixed in 1% formaldehyde for 15 min at room temperature. Cells were lysed with a bead beater (Biospec Products) and sonicated using a BioRuptor sonicating water bath (Diagenode) for 15 min (30-s duty cycle). For immunoprecipitations, H3K9me2 (m5.1.1 [48]), H3K9ac (Active Motif, catalog no. 39137), H3K14ac (Millipore, catalog no. 07-353), Flag M2 (Sigma, catalog no. F1804), and c-Myc (Santa Cruz, catalog no. 9B11) antibodies were incubated overnight with the lysate. After bead washes, samples were treated with Chelex resin, followed by incubation with proteinase K. Samples were diluted and used in quantitative PCRs (qPCRs).

### RNA analyses.

Reverse transcription-PCR (RT-PCR) of centromeric outer repeat transcripts and Northern analysis of centromeric siRNAs were performed as previously described (47, 49, 50).

### qPCRs.

ChIP and RT-PCR samples were quantified by qPCRs carried out with SYBR green on a LightCycler 480 instrument (Roche). The data were analyzed using Light Cycler 480 software 1.5 (Roche). For ChIP, enrichment was calculated as the ratio of target sequence to control sequence (*act1*^*+*^) in the immunoprecipitated sample over input lysate. For RT-PCR, quantification was relative to untreated dimethyl sulfoxide (DMSO) control using *act1*^*+*^/GAPDH as a negative region. Histograms represent mean and standard deviation for three biological replicates. Statistical significance was assessed by using the Student *t* test comparing compound-treated strains with untreated strains of the same genotype or mutant strains with the wild-type strain. Table 2 lists the primers used in the present study.

**TABLE 2:** Primers or probes used in this study

Primer or probe	Sequence (5′–3′)	
	Forward	Reverse
qPCR primers		
act1	CCCAAATCCAACCGTGAGAAGATG	CCAGAGTCCAAGACGATACCAGTG
kanMX	GGCCTGTTGAACAAGTCTG	TCCGACTCGTCCAACATC
cen(dg)	AATTGTGGTGGTGTGGTAATAC	GGGTTCATCGTTTCCATTCAG
mat	GTCCGAGGCAATACAACTTTGG	GGTTGACAGTAGGAGATATTTACAG
tlh	GGATAAGCCAATCATCGTTGAG	GTAGTTGACGCTCCTTGGAAG
Pc	CAGGTGCTTCAGCCAAATG	GCTAATTGTGACCAGGCAAG
Pi	CACTAAACCCCACTTGATGC	CGCTGACAGGTCGTAAAACTC
Mc	CCTGTTGGATGGGAATTCTG	AAAAGCATTAGGGGGTCTCG
eGFP	AGGGCTATGTGCAGGAGAGA	GGGTGGACAGGTAATGGTTG
amylase	TTCTGCTGCTTTCCCTCATT	CGAACAGGTGGACAATAGCA
GAPDH	ACCCAGAAGACTGTGGATGG	CACATTGGGGGTAGGAACAC
HDAC6	TAAGGAAATGACCACACCGA	CTGAGCAAGCACAGCCTTAG
HDAC10	TGGAGTGCTCCATCAAGAAG	TCTTATCTGCCCATCCATGA
Northern probes		
IK8	ATTCCTTTCTGAACCTCTCTGTTAT	
IK9	TTTGATGCCCATGTTCATTCCACTTG	
IK10	GGGAGTACATCATTCCTACTTCGATA	
snR58	GATGAAATTCAGAAGTCTAGCATC	

### Microarray analysis of gene expression.

Total RNA was extracted from S. pombe cells using the hot phenol method (51). RNA was purified with an RNeasy mini-kit (Qiagen) and tested for integrity on a 2200 Tape-station (Agilent). 150 nanograms of RNA was used for labeling and array hybridization using GeneChip S. pombe tiling 1.0FR microarrays according to the GeneChip Whole Target labeling and hybridization protocol (Affymetrix). The data were normalized with quantile normalization plus scaling using Tiling Analysis Software (TAS) 1.1 and imported into the Podbat browser program (52). Podbat was used to produce upregulated gene lists for the different treatments using settings for various fold changes and a *P* value of <0.05. GeneSpring GX software (Agilent Technologies) was used to compare the gene lists obtained for compound treatments with the gene lists for various S. pombe mutants present in an in-house database in the Ekwall laboratory. Cutoffs of 1.28 and 1.5 were used for the HMSI-1 and HMSI-2 treatments, respectively, to recover a comparable number of genes for both treatments. Biological duplicates were prepared and analyzed for each condition.

### Plant growth and histochemistry.

The transgenic Arabidopsis thaliana L5 line has been described elsewhere (53). Seeds were surface sterilized and sown on MS agar plates with 15 g/liter sucrose. Plates were cold treated for 2 days at 4°C. Seedlings were grown at 20°C under fluorescence white light (fluence rate of 40 to 60 μmol m^−2^ s^−1^) with a 16-h light/8-h dark photoperiod. At 12 days postgermination, seedlings were transferred to liquid MS in a 40-ml flask and, after 24 h, 10 μM HMS-I1, 10 μM HMS-I2, or DMSO was added. Compound treatment was repeated 3 days later, and samples were collected 2 days later. β-Glucuronidase (GUS) staining was performed as previously described (54) with minor modifications. Plant tissues were immersed in histochemical GUS staining buffer {100 mM NaPO_4_, 0.5 mM K_3_[Fe(CN)_6_], 0.5 mM K_4_[Fe(CN)_6_], 20% methanol, 0.3% Triton X-100, and 1 mg/ml X-Gluc (5-bromo-4-chloro-3-indolyl-β-d-glucuronic acid; pH 7)}, vacuum infiltrated under pressure for 10 min three times, and then incubated overnight at 37°C. Samples were washed several times with absolute ethanol until complete tissue clarification, incubated for 24 h in water, and stored in 50% glycerol. Images were taken using a Dotslide Olympus VS120.

### Transcriptional silencing assay in MEL cells.

Murine erythroleukemia (MEL) suspension cells were grown in Dulbecco modified Eagle medium (Gibco) with fetal bovine serum and penicillin-streptomycin at 37°C in the presence of each compound or DMSO solvent control. fluorescence-activated cell sorting (FACS) analysis of enhanced green fluorescent protein (eGFP) expression was performed with a Becton Dickinson FACS analyzer. For ChIP analysis, MEL cells were grown for 3 days in the presence of compounds and then fixed with 1% formaldehyde for 5 min at room temperature. Immunoprecipitated DNA was obtained with antibodies to H3K9me2 (Urano; Kimura 007) and H3K9me3 (Abcam; Kimura 008). Quantitation of ChIP was performed by qPCR as described above.

### Recombinant Clr3 protein production.

Using the MultiBac system (55), Clr3 was expressed in Sf9 insect cells as an N-terminal fusion of the codon optimized sequence to a multifunctional tag composed of OneStrep-Sumostar (OSS) (56), T7 tag, and a TEV site. The OSS-Clr3 protein was purified on a StrepTrap column (GE Healthcare), cleaved from the tag using tobacco etch virus (TEV) protease, and purified to homogeneity by gel filtration on Superdex 200 (GE Healthcare) in 10 mM HEPES (pH 7.6), 200 mM KCl, 1 mM dithiothreitol, and 50 mM magnesium acetate.

### *In vitro* HDAC assays.

Clr3 assays were performed with an HDAC fluorometric assay kit from BioVision (K330-100) according to the manufacturer's instructions. Recombinant human HDAC assays were performed by Reaction Biology (Malvern, PA). Compounds and purified recombinant HDACs were preincubated 20 min in assay buffer (50 mM Tris-HCl [pH 8.0], 137 mM NaCl, 2.7 mM KCl, and 1 mM MgCl_2_, 1 mg/ml bovine serum albumin, 1% DMSO), before the addition of 50 μM fluorogenic substrate [Arg-His-Lys-Lys(Ac)-AMC]. Reaction mixtures were incubated for 2 h at 30°C. The deacetylated substrate was then cleaved by trypsin, which released the fluorescent 7-amino-4-methylcoumarin. Fluorescent signals were read with an EnVision multilabel plate reader (Perkin-Elmer).

### siRNA knockdown of HDAC6 and HDAC10 in MEL cells.

Cells were transfected with sense and antisense small interfering RNA (siRNA) at a final concentration of 100 nM (HDAC6 sense, GGAGGAAGAUGAAGUGGAAuu; HDAC10 sense, GGACAAGCCUCCAGCAAAAuu) with DharmaFECT2 reagent. Cells were collected 48 h after transfection. Scrambled siRNAs were included as controls. Statistical significance was assessed by using the Student *t* test comparing HDAC6/10 siRNA with scrambled siRNA treatments.

### GEO accession number.

All microarray data are available under GEO accession number GSE57207.

## RESULTS

### Small-molecule inhibitors of fission yeast centromeric heterochromatin integrity.

**FIG 1:**
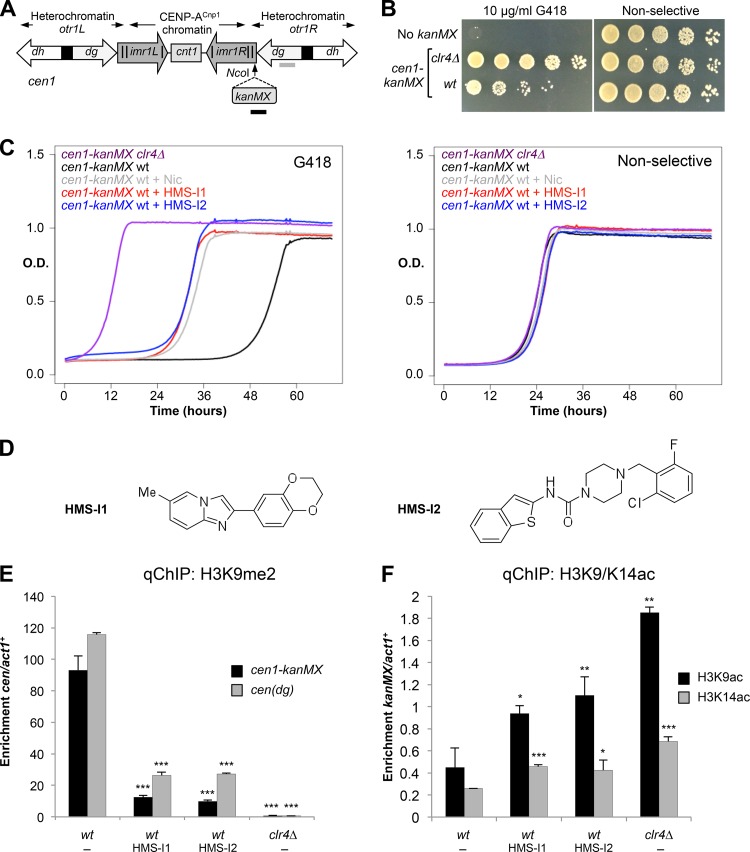
HMS-I1 and HMS-I2 disrupt centromeric heterochromatin integrity in fission yeast. (A) Schematic diagram showing the position of the *cen1 imr1R*(NcoI):*kanMX* (*cen1-kanMX* for short) reporter gene in centromere 1 relative to the *dg* and *dh* outer repeat (*otr*) elements, the innermost repeats (*imr*) and the central core (*cnt*). The position of heterochromatin and CENP-A^Cnp1^ chromatin domains is indicated. Locations of primers used in panel E below are indicated by the gray and black bars. *cen*(*dg*) primers amplify a sequence present in all centromeres. (B) Plating assay of the *cen1-kanMX* strain on YES plates with 10 μg/ml G418 or without G418 (nonselective). (C) Growth curves of *cen1-kanMX* cells in YES media with 5 μM HMS-I1, 5 μM HMS-I2, and 10 mM nicotinamide (Nic), with 10 μg/ml G418 or without G418 (nonselective). (D) Chemical structures of HMS-I1 and HMS-I2. (E) qChIP analysis of H3K9me2 levels associated with the *cen1-kanMX* insertion or *cen*(*dg*) repeats, relative to *act1*^*+*^. (F) qChIP analysis of H3K9ac and H3K14ac levels associated with the *cen1-kanMX* insertion, relative to *act1*^*+*^. In panels E and F, cells were grown in media containing 5 μM HMS-I1 and 5 μM HMS-I2, with 10 μg/ml G418. Error bars indicate standard deviations for three biological replicates. *, *P* < 0.05; **, *P* < 0.01; ***, *P* < 0.0001.

To screen for inhibitors of fission yeast heterochromatin integrity, we constructed a reporter strain harboring a dominant selectable kanamycin (*kanMX*) reporter gene within the heterochromatin of centromere 1 (*cen1*). The *kanMX* gene was inserted in the innermost repeats (*imr*) on the right side of *cen1* [*cen1-imr1R*(NcoI):*kanMX*, abbreviated as *cen1-kanMX*] (Fig. 1A). Expression of *kanMX* normally confers resistance to G418, but because in *cen1-kanMX* cells the reporter gene is silenced by surrounding heterochromatin, these otherwise wild-type cells exhibit sensitivity and grow poorly on plates containing G418. Disruption of heterochromatin by deletion of the gene encoding Clr4 methyltransferase (*clr4*Δ) allowed expression of *cen1-kanMX* and resistance to G418 and thus increased growth on G418 medium (Fig. 1B). Microwell-based liquid culture growth assays, performed with or without G418 on *cen1-kanMX* cells, enabled the reproducible detection of G418 resistance in *clr4*Δ cells compared to wild-type (*wt*) cells by continual monitoring of optical density. Addition of the known Sirtuin HDAC inhibitor nicotinamide also resulted in increased growth of wild-type cells in the presence of G418 (Fig. 1C). This validated *cen1-kanMX* reporter strain was subsequently used to screen 1,570 compounds that had been previously selected for chemical diversity and bioactivity (57). Two compounds, 2-(2,3-dihydro-1,4-benzo[*b*][1,4]dioxin-6-yl)-6-methylimidazo[1,2-*a*]pyridine and *N*-(1-benzo[*b*]thiophen-2-yl)-4-(2-chloro-6-fluorobenzyl)piperazine-1-carboxamide, here referred to as HMS-I1 and HMS-I2, respectively (*h*eterochromatin-*m*ediated *s*ilencing *i*nhibitors 1 and 2), were identified as reproducible hits that increased the resistance of wild-type *cen1-kanMX* cells to G418 to a similar extent as nicotinamide (Fig. 1C and D).

To our knowledge, biological activity for HMS-I1 and HMS-I2 has not been reported previously. Although HMS-I1 and HMS-I2 could conceivably fit into a common pharmacophore model, their structural disparities precluded straightforward conclusions. For instance, the imidazopyridine and piperazine carboxamide cores of HMS-I1 and HMS-I2, respectively, feature different numbers and placement of hydrogen bond donors/acceptors, while their lipophilic flanking groups differ in size and shape. HMS-I1 and HMS-I2 lack compelling structural similarities to well-established inhibitors of chromatin-modifying enzymes such as HDAC inhibitors (58, 59). We note that HMS-I1 and HMS-I2 do not contain canonical zinc chelating moieties possessed by type I and II HDAC inhibitors: HMS-I1 cannot form a chelate due to a lack of heteroatoms disposed in a 1,4 relationship and the conceivable chelation modes for HMS-I2 are electronically and sterically suboptimal. Taken together, such structural considerations suggest that HMS-I1 and HMS-I2 may be acting via a novel mechanism.

A hallmark of centromeric heterochromatin is the presence of methylated histone H3K9 (H3K9me) chromatin over the centromeric repeats and inserted marker genes. Disruption of centromeric heterochromatin results in reduced levels of H3K9me and loss of silencing of inserted marker genes (28, 47, 60). Quantitative ChIP (qChIP) analyses on cells grown under G418 selection revealed that HMS-I1 and HMS-I2 caused substantial loss of H3K9me2 from both the *kanMX* gene and the centromeric repeat elements (Fig. 1E). Importantly, the qChIP assay monitored H3K9me2 associated with the 18 copies of *otr/dg* centromere repeats distributed over the three centromeres. HMS-I1 and HMS-I2 thus altered the status of H3K9 methylation across all three centromeres, demonstrating that the compounds affect heterochromatin throughout the nucleus and not just at the selected *cen1-kanMX* locus. Neither compound induced a complete loss of H3K9me2, as observed in cells lacking Clr4 H3K9 methyltransferase (*clr4*Δ) (Fig. 1E). Growth of *cen1-kanMX* cells in the presence of HMS-I1 or HMS-I2, but in the absence of G418 selection, had less dramatic effects since the H3K9me2 levels were lower on *kanMX* but not on the native outer repeat sequences (data not shown and see Fig. 3).

Histone hypoacetylation is also a hallmark of heterochromatin, and an increase in histone lysine acetylation is expected to accompany the loss of centromeric marker gene silencing. Consistently, we observed an increase in H3K9 and H3K14 acetylation on *cen1-kanMX* in cells treated with HMS-I1 and HMS-I2 grown in the presence of G418 (Fig. 1F). We conclude that HMS-I1 and HMS-I2 increase the G418 resistance of *cen1-kanMX* cells by general disruption of centromeric heterochromatin and concomitant elevated expression of the embedded *kanMX* resistance gene.

### HMS-I1 and HMS-I2 inhibit events downstream of RNAi in chromatin modification and heterochromatin formation.

**FIG 2:**
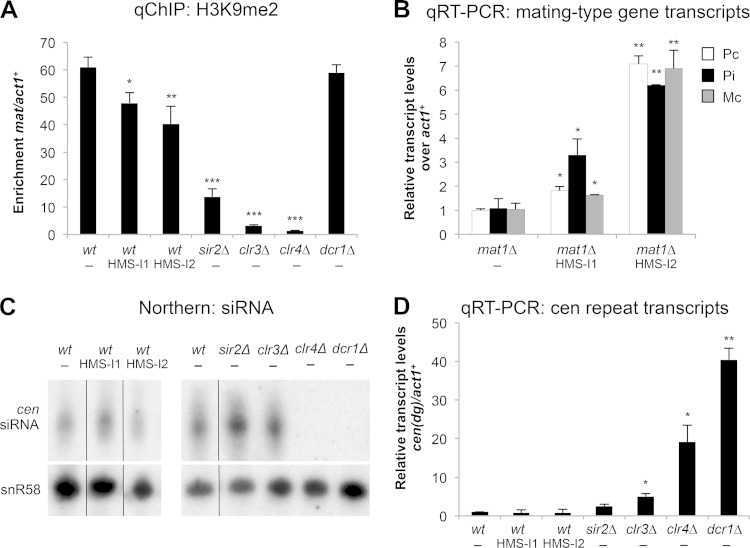
HMS-I1 and HMS-I2 phenocopy loss of Clr3 or Sir2. (A) qChIP analysis of H3K9me2 levels associated with the silent *mat2-mat3* region of the mating-type locus (*mat*), relative to *act1*^*+*^. Error bars indicate standard deviations from three biological replicates. (B) Quantification of mating-type gene transcripts (Pc, Pi, and Mc), relative to *act1*^*+*^ transcript levels and normalized to relative levels in untreated *mat1*Δ cells. Error bars indicate standard deviations from two biological replicates. (C) Northern blot analysis of enriched centromeric siRNAs. snoRNA58 (snR58) was used as a loading control. (D) Quantification of *cen*(*dg*) relative to *act1*^*+*^ transcript levels and normalized to relative levels in wild-type cells. Error bars indicate standard deviations for three biological replicates. *, *P* < 0.05; **, *P* < 0.01; ***, *P* < 0.0001. HMS-I1 and HMS-I2 were used at a concentration of 5 μM in panels A to D. The media contained 10 μg/ml G418 in panels A, C, and D.

RNAi is required for both the establishment and the maintenance of centromeric heterochromatin in fission yeast (61–63). In contrast, following RNAi-triggered establishment of heterochromatin domains over the *mat2-mat3* region of the mating-type locus or adjacent to telomeres, RNAi is not continuously required to maintain heterochromatin since alternative pathways propagate heterochromatin at these locations (64–67). ChIP analyses showed that, as expected, H3K9me2 was lost at *mat2-mat3* and telomeres in cells that lacked Clr4 H3K9 methyltransferase, or the HDACs Clr3 and Sir2, but was retained in cells that lack RNAi due to deletion of Dcr1. H3K9me2 levels were also consistently reduced at both mating-type and subtelomeric regions when *cen1-kanMX* cells were incubated with HMS-I1 or HMS-I2 and selected for G418 resistance (Fig. 2A and data not shown).

Heterochromatin-mediated silencing at the mating-type locus ensures that the silent mating-type cassettes encoding plus (P) or minus (M) information (*mat2-P* and *mat3-M*) cannot be expressed. These cassettes must normally be recombined into the active *mat1* expression site to escape the powerful repressive heterochromatin environment of the silent loci and allow expression. Mutations in CLRC (Clr4-Rik1-Cul4) complex and HDAC components are known to alleviate transcriptional silencing at the *mat2-P* and *mat3-M* loci (26). Strikingly, treatment of a sterile fission yeast strain lacking the *mat1* expression site (i.e., a *mat1*Δ strain that lacks the *kanMX* reporter gene) with HMS-I1 and HMS-I2 resulted in the expression of Pc/Pi and Mc transcripts from the silent *mat2-P* and *mat3-M* cassettes, respectively (Fig. 2B). Thus, similar to the deletion of HDAC and CLRC components, HMS-I1 and HMS-I2 disrupt the robust form of heterochromatin-mediated silencing that normally ensures that *mat2-P* or *mat3-M* are not transcribed. From these results we concluded that both HMS-I1 and HMS-I2 must interfere with heterochromatin formation downstream of RNAi.

In cells with defective RNAi or Clr4, noncoding outer repeat transcripts accumulate as they are no longer processed by RNAi to siRNA and transcriptional silencing is reduced. In contrast, cells that lack either Clr3 or Sir2 exhibit reduced H3K9 methylation levels on centromeric repeats but retain high siRNA levels with only a slight increase in centromeric transcripts (15, 16, 62). These phenotypic differences allow the mechanism of action of specific mutants or inhibitors to be distinguished. Despite the reduced levels of H3K9me2 detected on centromere repeats in wild-type cells grown in the presence of HMS-I1 or HMS-I2 and G418, these cells retained high levels of centromeric siRNA, while centromeric repeat transcripts remained low (Fig. 2C and D). Hence, HMS-I1 or HMS-I2 treated cells resemble *clr3*Δ and *sir2*Δ cells. These results demonstrate that HMS-I1 and HMS-I2 phenocopy the loss of Clr3 and Sir2 and suggest that both compounds might interfere with either Clr3- or Sir2-related functions.

### The Clr3 HDAC is a putative target of HMS-I1 and HMS-I2.

**FIG 3:**
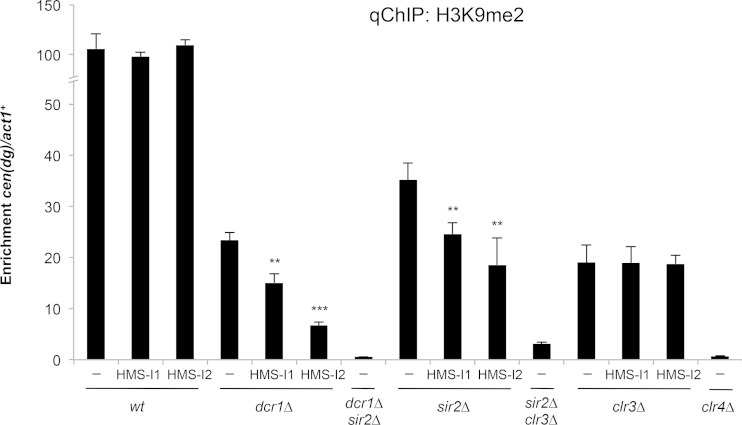
Clr3 is a putative target of HMS-I1 and HMS-I2. qChIP analysis of H3K9me2 levels associated with centromeric repeats [*cen*(*dg*)], relative to *act1*^*+*^. Cells were grown in the presence of 5 μM HMS-I1 or HMS-I2 and without G418 selection. Error bars indicate standard deviations for three biological replicates. *, *P* < 0.05; **, *P* < 0.01; ***, *P* < 0.0001.

Epistasis analysis has shown that RNAi operates in a parallel pathway to the HDACs Clr3 and Sir2 to maintain heterochromatin integrity at centromeres (61, 62, 68). Cells devoid of an RNAi component such as Dcr1, or either the Clr3 or Sir2 HDACs, retain significant levels of H3K9me on centromeric repeats. However, the removal of both Dcr1 and Clr3 or Sir2 reduces H3K9me to much lower levels than that observed in *dcr1*Δ, *clr3*Δ or *sir2*Δ cells alone (62, 68). To determine whether HMS-I1 and HMS-I2 might exacerbate the phenotype of cells lacking Dcr1, Clr3 or Sir2, we compared the levels of H3K9me2 on centromeric repeats in wild-type, *dcr1*Δ, *clr3*Δ, or *sir2*Δ cells grown in the presence or absence of either compound, all in the absence of G418 selection. HMS-I1 and HMS-I2 exhibited strong chemical-genetic interactions with *dcr1*Δ and *sir2*Δ, since a greater reduction in the level of H3K9me2 associated with centromeric repeats was observed in the presence of each compound but not with *clr3*Δ (Fig. 3). HMS-I1 and HMS-I2 therefore behaved as chemical mimetics of the *clr3*Δ mutation in terms of interactions with either *dcr1*Δ or *sir2*Δ (62, 68). These chemical-genetic interactions, coupled with the observation that neither compound further reduced H3K9me2 levels on centromere repeats in *clr3*Δ cells, imply that both HMS-I1 and HMS-I2 interfere with the function of either the HDAC Clr3 and/or associated proteins in the SHREC complex.

### HMS-I1 and HMS-I2 mimic the effect of SHREC mutations on gene expression.

**FIG 4:**
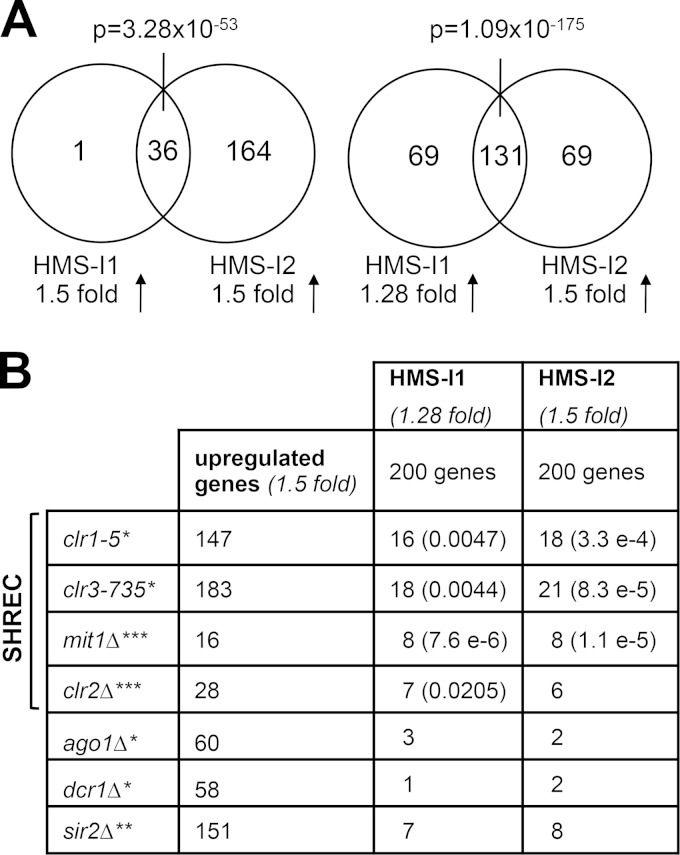
HMS-I1 and HMS-I2 mimic the effect of SHREC mutations on gene expression. (A) Overlap between gene sets upregulated in cells grown in the presence of HMS-I1 and HMS-I2. (B) Expression profiles of cells grown in the presence of HMS-I1 or HMS-I2 compared to the indicated SHREC mutants. The *P* value (hypergeometric probabilities) of the overlap between gene lists is indicated in cases where a statistical significance was observed. Genes upregulated in cells that lack RNAi (*ago1*Δ and *dcr1*Δ) or the Sir2 HDAC (*sir2*Δ) were also compared but showed no significant overlap with those upregulated by HMS-I1 and HMS-I2 treatment. *, data from Hansen et al. (69); **, data from Wiren et al. (18); ***, data from the present study (see Data set S1 in the supplemental material). Cells were grown in YES medium with 5 μM HMS-I1 or HMS-I2 and 10 μg/ml G418 in panels A and B. Comparisons in panels A and B are restricted to annotated genes present in the Ekwall laboratory in-house database.

Previous genome-wide expression profiles showed that the Clr3 HDAC is required to repress expression of a broad cohort of genes (183 genes > 1.5-fold upregulated in *clr3-735* [69]). Clr3 has been shown to associate with Clr1, Clr2, and Mit1 within the repressive SHREC complex, and all four components contribute to heterochromatin integrity (15). If HMS-I1 and HMS-I2 interfere with Clr3 or SHREC function as predicted, then each compound should cause induction of a gene set that overlaps with that upregulated in SHREC mutant cells. In cells grown in the presence of compounds and G418, HMS-I1 and HMS-I2 increased the expression of 37 and 200 genes by >1.5-fold, respectively. Significant overlap was evident between the sets of genes affected by HMS-I1 and HMS-I2: 36 of the 37 genes upregulated 1.5-fold by HMS-I1 were also upregulated 1.5-fold by HMS-I2 (*P* = 3.28 × 10^−53^) and 131 of the 200 genes upregulated 1.28-fold by HMS-I1 were also upregulated 1.5-fold by HMS-I2 (*P* = 1.09 × 10^−175^) (Fig. 4A; see Data set S1 in the supplemental material). Compared to expression profiles for *clr1-5*, *clr2*Δ, *clr3-735*, and *mit1*Δ strains (69) (see also Data set S1 in the supplemental material), we observed significant overlap between genes derepressed in the SHREC mutants and HMS-I1 or HMS-I2 treated cells (Fig. 4B). No significant overlap was detected when we compared genes upregulated by HMS-I1 or HMS-I2 with those increased in cells that lack RNAi components (*ago1*Δ and *dcr1*Δ; Fig. 4B). Previous analyses showed that Clr3 and Sir2 mediate the repression of an overlapping set of genes (18), which explains the few genes whose expression increases after exposure to HMS-I1 or HMS-I2 and that are also upregulated in *sir2*Δ cells (Fig. 4B). However, the overlap between genes upregulated by HMS-I1 and HMS-I2 and those upregulated in *sir2*Δ cells is not significant. Based on this analysis of global gene expression, we conclude that the SHREC complex is a likely target for the inhibitory activity of both HMS-I1 and HMS-I2.

### HMS-I1 and HMS-I2 reduce *in vitro* Clr3 HDAC activity.

**FIG 5:**
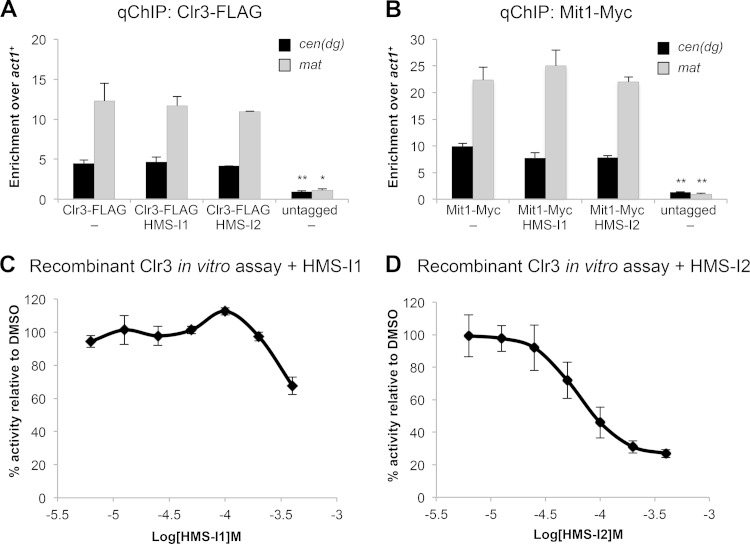
HMS-I1 and HMS-I2 inhibit Clr3 HDAC activity *in vitro* without affecting recruitment of SHREC at heterochromatin. (A and B) qChIP analysis of Clr3 (Clr3-3XFLAG) (A) and Mit1 (Mit1-13xmyc) (B) recruitment at centromeric repeats [*cen*(*dg*)] and at the silent *mat2-mat3* region of the mating-type locus (*mat*), relative to *act1*^*+*^. Cells were grown in the presence of 5 μM HMS-I1 or HMS-I2. Error bars indicate the standard deviations of three biological replicates. *, *P* < 0.05; **, *P* < 0.01; ***, *P* < 0.0001. (C and D) Fluorescence-based deacetylation assays were used to detect inhibition of recombinant S. pombe Clr3 HDAC activity *in vitro* at the indicated concentrations of HMS-I1 (C) and HMS-I2 (D). Error bars indicate standard deviations for three replicates.

The genetic analyses described above indicate that HMS-I1 and HMS-I2 impede the activity of the fission yeast SHREC complex, which contains the type II HDAC Clr3. We first assessed whether HMS-I1 and HMS-I2 acted by preventing the recruitment of SHREC components at heterochromatin. ChIP analyses showed that neither HMS-I1 nor HMS-I2 affected the recruitment of either the Clr3 HDAC or the Mit1 chromatin remodeling components of SHREC to mating-type or centromeric heterochromatin (Fig. 5A and B). To determine whether these compounds exert their effect on SHREC function through inhibition of Clr3 catalytic activity, we performed *in vitro* fluorescence-based HDAC assays. HMS-I1 and HMS-I2 reproducibly reduced the activity of Clr3 by 40 and 80%, respectively (Fig. 5C and D). Under these assay conditions, HMS-I2 inhibits Clr3 to a greater extent than HMS-I1, a finding consistent with the greater reduction in H3K9 methylation observed in HMS-I2-treated *sir2*Δ and *dcr1*Δ cells and the greater increase in gene expression caused by HMS-I2 (see Fig. 3 and 4). Collectively, these data suggest that HMS-I1 and HMS-I2 are inhibitors of Clr3 HDAC activity.

### HMS-I1 and HMS-I2 disrupt transgene silencing in multicellular eukaryotes.

**FIG 6:**
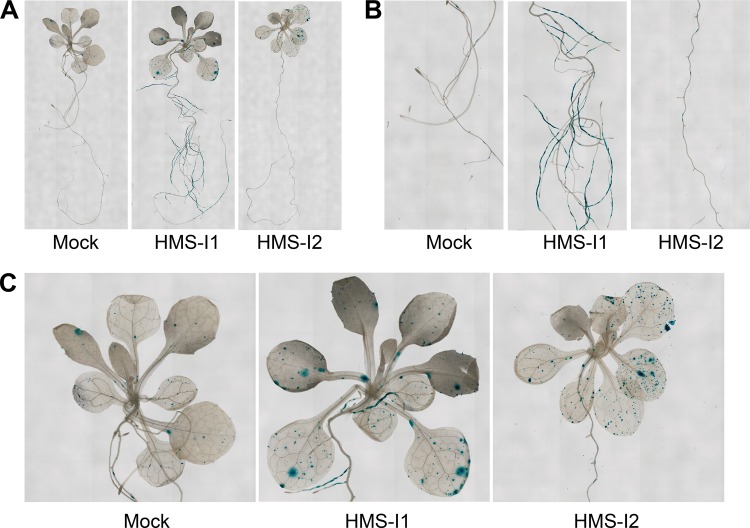
HMS-I1 and HMS-I2 alleviate transcriptional silencing of a GUS transgene in Arabidopsis L5 seedlings. (A) Histochemical staining of A. thaliana L5 transgenic seedlings for β-glucuronidase (GUS) activity after incubation with 10 μM HMS-I1, 10 μM HMS-I2, or solvent control. (B) Magnified view of root tissue. (C) Magnified view of leaf tissue.

To assess whether HMS-I1 and HMS-I2 might also disrupt heterochromatin integrity in multicellular eukaryotes, we tested for the effects on gene silencing in plants and mammalian cells. We first tested whether HMS-I1 and HMS-I2 could alleviate reporter gene silencing in the Arabidopsis thaliana L5 transgenic line, which contains a transcriptionally silenced GUS transgene (53). HMS-I1 caused an increase in the GUS reporter expression in the root and leaf tissue, whereas HMS-I2 alleviated silencing in the leaf tissue only (Fig. 6). Transcriptional gene silencing mutants reactivate the expression of GUS, whereas RNAi mutants do not (70), indicating that, as in fission yeast, HMS-I1 and HMS-I2 mediate their effects in Arabidopsis independently of RNAi.

**FIG 7:**
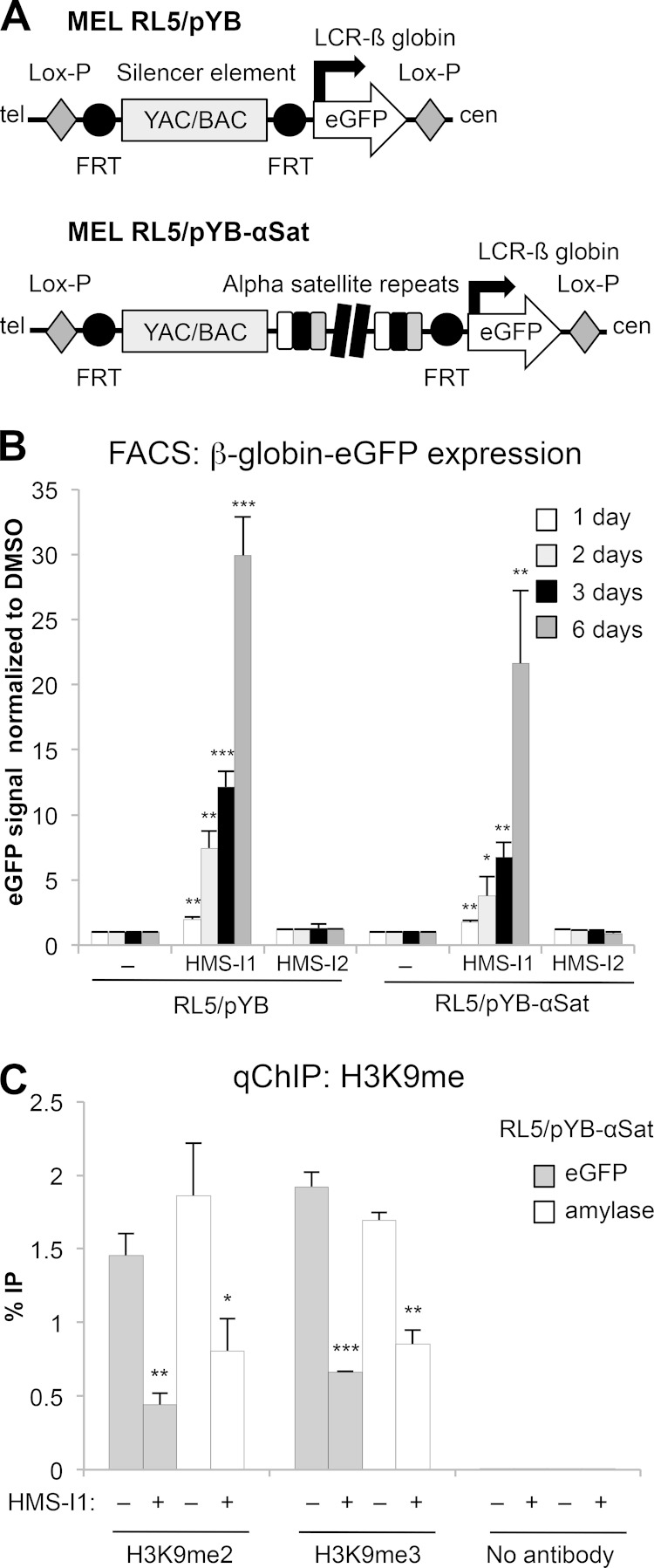
HMS-I1 disrupts heterochromatin mediated transcriptional silencing in murine erythroleukemia (MEL) cells. (A) Schematic diagram of the β-globin/eGFP reporter inserted with (MEL RL5/pYB-αSat) or without (MEL RL5/pYB) adjacent α-satellite arrays at the RL5 locus in MEL cells (71). (B) FACS analysis to monitor eGFP expression by fluorescence of RL5/pYB or RL5/pYB-αSat MEL cells. Fluorescence was normalized relative to untreated DMSO controls, after consecutive days of growth in medium containing 10 μM HMS-I1 or 10 μM HMS-I2. Error bars indicate the standard deviations from three biological replicates. (C) qChIP analysis of H3K9me2 and H3K9me3 levels associated with the RL5/pYB-αSat β-globin–eGFP reporter gene and the endogenous mouse amylase gene after 3 days incubation with or without 10 μM HMS-I1. Error bars indicate standard deviations for three biological replicates. *, *P* < 0.05; **, *P* < 0.01; ***, *P* < 0.0001.

We next assessed the effect of the compounds on reporter gene silencing in MEL cells. A β-globin gene promoter-driven eGFP reporter is known to be silenced and coated in repressive H3K9 methylated chromatin at the RL5 locus in these MEL cells when vector backbone bacterial DNA is inserted upstream of the transgene (designated RL5/pYB) (Fig. 7A) (71). In addition, the inclusion of an array of human α-satellite repeats upstream of the reporter (designated RL5/pYB-αSat) (Fig. 7A) bolsters eGFP silencing and H3K9me levels across the transgene (71). We utilized both RL5/pYB and RL5/pYB-αSat MEL cell lines to determine whether HMS-I1 or HMS-I2 interfere with the integrity of heterochromatin at the reporter locus. FACS analysis of the RL5/pYB and RL5/pYB-αSat cell lines showed that eGFP expression increased over time with HMS-I1 but not with HMS-I2 (Fig. 7B). Thus, growth in the presence of HMS-I1 overcomes the strong silencing mediated by α-satellite repeats in MEL cells.

To determine whether HMS-I1 also alters H3K9 methylation levels associated with silent transgenes in multicellular eukaryotes, we performed ChIP analysis with the RL5/pYB-αSat cell line, for which the observed effect on silencing was the strongest. A substantial decrease in both H3K9me2 and H3K9me3 levels associated with the eGFP reporter occurred after 3 days of incubation with HMS-I1. Furthermore, a reduction in H3K9 methylation was also detected on the endogenous mouse amylase gene (Fig. 7C) that is normally repressed in MEL cells (72). Thus, HMS-I1 can disrupt heterochromatin associated with endogenous repressed genes, in addition to that formed by mammalian repetitive elements over the β-globin/eGFP reporter.

**FIG 8:**
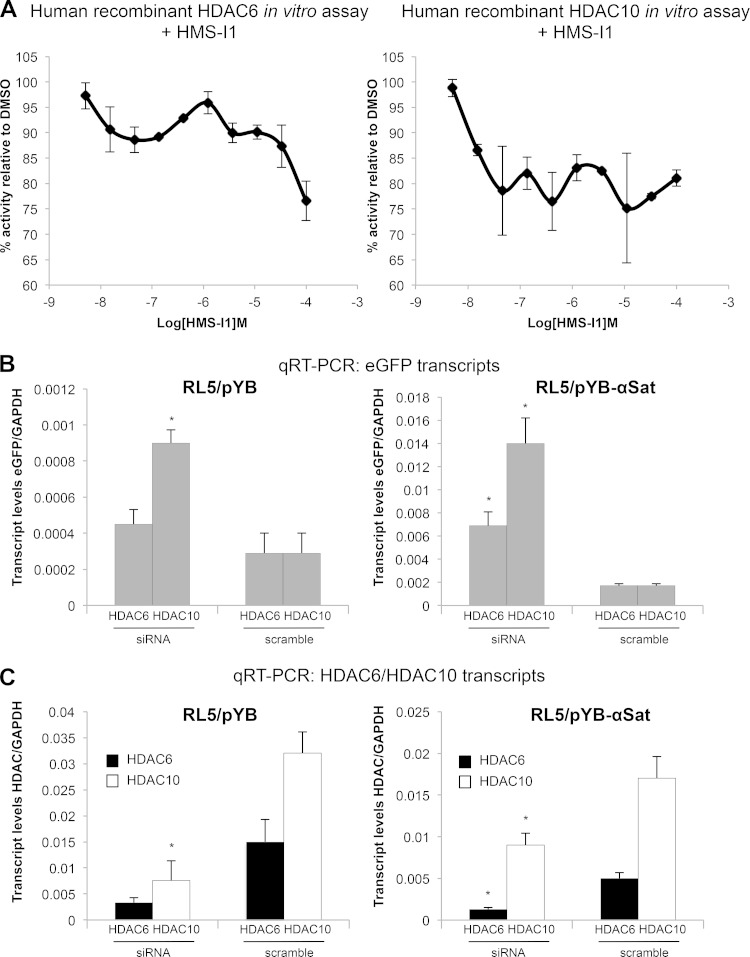
HMS-I1 reduces the activity of mammalian HDAC6 and HDAC10 *in vitro*. (A) Fluorescence-based deacetylation assays were used to detect inhibition of recombinant human HDAC6 and HDAC10 activity *in vitro* at the indicated concentrations of HMS-I1. Error bars indicate the standard deviations from two replicates. (B) Quantification of eGFP transcripts relative to GAPDH (glyceraldehyde-3-phosphate dehydrogenase) transcript levels after siRNA knockdown of HDAC6 and HDAC10 in MEL cells with a β-globin/eGFP reporter inserted with (RL5/pYB-αSat) or without (RL5/pYB) adjacent α-satellite arrays at the RL5 locus. Error bars indicate the standard deviations from three biological replicates. (C) Quantification of HDAC6 and HDAC10 transcripts relative to GAPDH transcript levels after siRNA knockdown of HDAC6 and HDAC10 in RL5/pYB and RL5/pYB-αSat MEL cells. Error bars indicate standard deviations for three biological replicates. *, *P* < 0.05.

In S. pombe, our data suggested that the type II HDAC Clr3 is a target of HMS-I1. Although the identity of HMS-I1 target(s) in mammalian cells remains to be defined *in vivo*, an *in vitro* HDAC assay performed with a panel of human recombinant enzymes indicated that HMS-I1 reduced the activity of the type IIb HDACs, HDAC6 and HDAC10 (Fig. 8A). Consistent with inhibition of these HDACs, siRNA knockdown of either HDAC6 or HDAC10 expression resulted in increased β-globin–eGFP transcript levels in both RL5/pYB and RL5/pYB-αSat MEL cell lines compared to scrambled siRNA controls (Fig. 8B and C). Collectively, these results suggest that HMS-I1, and possibly also HMS-I2, derepress transcriptionally silent heterochromatin in evolutionarily distant species through inhibition of conserved HDACs.

## DISCUSSION

Heterochromatin is tightly associated with transcriptional silencing in most eukaryotes and is particularly prevalent over repetitive elements, where it prevents inappropriate recombination events and genome rearrangements (3). The aberrant silencing of genes through unscheduled heterochromatin formation is known to contribute to several human diseases, including acute myeloid leukemia and Friedreich's ataxia (73, 74). Moreover, the manipulation of transcriptional silencing has been used to improve crop-plant produce and yields (75). Since RNAi-related mechanisms establish sequence-specific heterochromatin in many eukaryotes, we exploited the well-understood process of heterochromatin formation in fission yeast to develop a novel screen for small-molecule modulators of heterochromatin integrity. Collectively, our analyses indicate that HMS-I2 alleviates heterochromatin-mediated gene silencing in both fungal and plant systems but not MEL cells, whereas HMS-I1 abrogates silencing across the fungal, plant, and animal kingdoms. Both HMS-I1 and HMS-I2 were observed to inhibit the *in vitro* activity of fission yeast class II Clr3 HDAC, whereas only HMS-I1 inhibited the human subclass IIb enzymes, HDAC6 and HDAC10. Phylogenetic analysis indicates that human HDAC6 and HDAC10 are closely related to Clr3 (76), with a similar domain architecture of the HDAC catalytic domain followed by a conserved C-terminal domain. Although the major substrate of HDAC6 has been reported to be acetylated α-tubulin (77), this enzyme has also been implicated in chromatin regulation and could play a direct role in histone deacetylation (78). HDAC10 possesses HDAC activity and has been implicated in autophagy and cancer (79, 80). Therefore, our cross-species comparisons suggest that type II HDAC enzymes may be conserved targets of HMS-I1 and potentially also HMS-I2.

Inspection of the chemical structure of HMS-I1 and HMS-I2 suggests that their mechanism of action is likely to differ from that of canonical HDAC inhibitors, particularly as neither HMS-I1 or HMS-I2 have obvious zinc-chelating characteristics. HMS-I1 and HMS-I2 may therefore represent a novel class of HDAC inhibitor. We note that while *in vitro* HDAC inhibition assays suggest that HMS-I1 and HMS-I2 directly target HDAC activity, it remains formally possible that these compounds also affect heterochromatin integrity *in vivo* by interfering with other activities and/or protein interactions within HDAC complexes. A detailed structure-activity relationship (SAR) analysis will be required to improve potency and further delineate the mechanism of HMS-I1 and HMS-I2 action. Regardless, our discovery of HMS-I1 and HMS-I2 as panspecies inhibitors of heterochromatin-mediated transcriptional gene silencing demonstrates the striking functional conservation of the silencing machinery through eukaryotic evolution. HMS-I1 and HMS-I2 should serve as new chemical probes for dissection of silencing functions in various species and represent possible candidates for early stage drug development in diseases that result from inappropriate gene silencing events.

## Supplementary Material

Supplemental material
