# Supplementary material for: Panspecies Small-Molecule Disruptors of Heterochromatin-Mediated Transcriptional Gene Silencing
Source: Mol Cell Biol. 2015 Jan 23;35(4):662–74. doi: 10.1128/MCB.01102-14 (PMC4301722; doi:10.1128/MCB.01102-14)
Supplement: Supplemental material [file supp_35_4_662__index.html]

Supplemental material 

# Panspecies Small-Molecule Disruptors of Heterochromatin-Mediated Transcriptional Gene Silencing

## Supplemental material

**Files in this Data Supplement:**

- Supplemental file 1 -

  Data set S1 (*S. pombe* genes up- and downregulated in HMS-I1-treated cells)

  XLSX, 127K
